# A novel redox/pH dual-responsive and hyaluronic acid-decorated multifunctional magnetic complex micelle for targeted gambogic acid delivery for the treatment of triple negative breast cancer

**DOI:** 10.1080/10717544.2018.1486472

**Published:** 2018-10-18

**Authors:** Mang Mang Sang, Fu Lei Liu, Yang Wang, Ren Jie Luo, Xiao Xian Huan, Ling Fei Han, Zhong Tao Zhang, Feng Feng, Wei Qu, Wenyuan Liu, Feng Zheng

**Affiliations:** a Key Laboratory of Drug Quality Control and Pharmacovigilance, Ministry of Education, China Pharmaceutical University, Nanjing, People’s Republic of China;; b Department of Pharmaceutical Analysis, China Pharmaceutical University, Nanjing, People’s Republic of China;; c Department of Natural Medicinal Chemistry, China Pharmaceutical University, Nanjing, People’s Republic of China

**Keywords:** Magnetic, redox/pH dual-responsive, hyaluronic acid, gambogic acid, TNBC

## Abstract

Gambogic acid (GA) is a naturally derived potent anticancer agent with extremely poor biocompatibility. In the present study, a novel of redox/pH dual-responsive multifunctional magnetic complex micelle (sPEG/HA/CSO-SS-Hex/Fe_3_O_4_/GA), which consisted of a reducible hexadecanol-modified chitosan oligosaccharide polymer micelle (CSO-SS-Hex) coated with hyaluronic acid (HA) and DCA grafted sheddable PEG-PLL (sPEG) copolymers and loaded with gambogic acid (GA) and Fe_3_O_4_ nanoparticles were developed for parenteral delivery for the treatment of triple negative breast cancer (TNBC). The *ex vivo* study showed that the sPEG shielded cationic HA/CSO-SS-Hex/Fe_3_O_4_/GA core at physiological pH but quickly shed off to re-expose the core due to its charge reversible property. The sPEG/HA/CSO-SS-Hex/Fe_3_O_4_/GA micelles effectively facilitated tumor-targeted GA delivery by HA, which is a targeting ligand for CD44 receptor of TNBC cells, meanwhile increase GA uptake at the acidic condition but diminished the drug uptake at neutral pH. The *in vitro* cellular uptake study and *in vivo* biodistribution and antitumor activity of the formulations were determined, all results showed that the complex micelle enhanced TNBC tumor cellular uptake and fast drug release due to the combined effect of magnet targeting, CD44 receptor-mediated internalization and redox/pH dual-responsive drug release. Hence, tumor-targeted delivery of GA with redox/pH dual-responsive multifunctional magnetic complex micelle sPEG/HA/CSO-SS-Hex/Fe_3_O_4_/GA might have potential implications for the chemotherapy of TNBC.

## Introduction

Breast cancer, especially triple-negative breast cancer (TNBC) which do not express any of the three markers of estrogen receptor (ER), progesterone receptor (PR) and human epidermal growth factor receptor 2 (HER2), is the most common cancer and the main cause of cancer-related mortality among women worldwide (Coley, 2008; Brewster et al., 2014; Palmer et al., 2014). Unlike other breast cancer cases, triple-negative breast cancer patients have little opportunity for choosing targeted therapy, therefore, normally receive systemic chemotherapy that has inevitable adverse effects (Gelmon et al., 2012; Brewster et al., 2014). Although the development of adjuvant chemotherapy has reduced the risk of death of TNBC patients by approximately 30% (Carey et al., 2007), there has been limited progress in incorporating additional systemic therapies for TNBC (Turner & Reis-Filho, 2013). Therefore, there is an urgent need to develop new drugs or novel formulation strategy to potentiate the therapeutic outcome to combat TNBC.

Gambogic acid (GA) is a recently explored polyprenylated xanthene, which is the main small molecule active ingredient of gamboge resin that is exuded from the Garcinia hanburyi tree in Southeast Asia has multiple therapeutic actions and was been used for hundreds of years in China (Auterhoff et al., 1962; Wang et al., 2013). Both *in vitro* and *in vivo* studies have demonstrated that GA has significant anticancer activity, against numerous types of human cancer, including prostate, gastric carcinoma, hepatocarcinoma, lung cancer, and breast carcinoma (Yang et al., 2007; Qi et al., 2008; Doddapaneni et al., 2016). It also has been approved for phase-II clinical trials by the Chinese Food and Drug Administration (Gu et al., 2009). But the poor water solubility (<5 ppm) and short biological half-life (less than 1 hour in dogs and less than 20 minutes in rats) (Liu et al., 2017) were proved to the major hurdle for GA clinical application.

To achieve successful TNBC-targeted GA therapy, we must overcome two main problems caused by biological barriers, one is how to specifically target to the tumor microenvironment of TNBC and the another one is how to facilitate GA uptake and cytosolic releasing (Price & Chen, 2014). Hybrid multifunctional complex micelles, such as the CPT-loaded FA-CLC/SPIO (Chen et al., 2015), DOX-loaded PCL-SS-PDMAEMA/Fe_3_O_4_ (Wang et al., 2016) and PTX/MNPs/QDs@Biotin-PEG-PCDA (Cheng et al., 2011) are usually composed of stimuli-responsive amphiphilic polymers with functions of effectively targeting the chemotherapeutic reagents to tumor cells, meanwhile, reducing toxicity to normal tissues. PEG-sheddable, mannose-modified poly (-lactic-co-glycolic acid) (PLGA) nanoparticle platform that can efficiently target to the tumor cell via mannose-mannose receptor recognition after acid-sensitive PEG shedding in the acidic tumor microenvironment. Thus, hybrid multifunctional micelles have provided a new strategy for the tumor targeted and accurately released of drugs in tumor microenvironment.

**Scheme 1.**
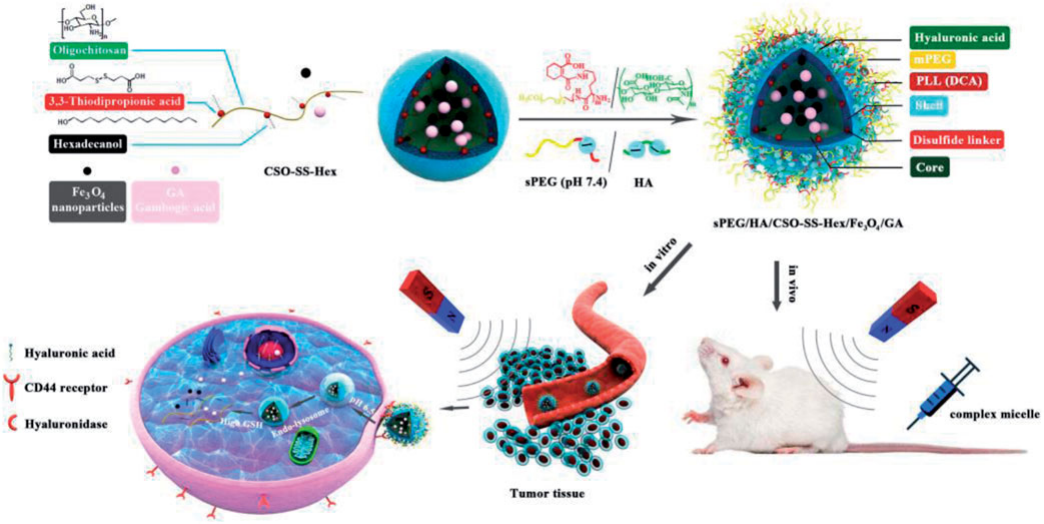
Scheme of sPEG/HA/CSO-SS-Hex/Fe_3_O_4_/GA micelle construction and magnetism-enhanced EPR trafficking pathway. The cellular trafficking pathway includes receptor-meditated cellular internalization, redox and pH triggered micelle disassembly and drug release.

We previously reported hybrid multifunctional complex micelles based on hyaluronic acid-decorated redox-responsive magnetic complex micelle (HA/CSO-SS- Hex/Fe_3_O_4_/PTX) as a versatile and efficient delivery system for drug (Sang et al., 2018). However, previous studies had two drawbacks: the nanoparticles with high positive surface charge and no *in vivo* research. Herein, redox/pH dual-responsive and magnetic targeted hybrid multifunctional complex micelles (sPEG/HA/CSO-SS-Hex/Fe_3_O_4_/GA) have been developed for tumor microenvironment targeted GA delivery and anti-cancer therapy. As shown in Scheme 1, the core of complex micelle is a reducible copolymer (Guo et al., 2015; Wang et al., 2017c) based on coupling CSO to disulfide-containing carboxylic acid (CSO-SS-Hex). Then, the Fe_3_O_4_ nanoparticles (Wang et al., 2017b) and GA were encapsulated into the core of the micelles. As a targeted ligand of CD44, HA was coated on the surface of micelles *via* electrostatic absorption. The obtained magnetic complex micelle could accumulate in tumor issue and be selectively taken up by tumor cells with the help of magnetism-enhanced EPR and CD44-receptor-mediated endocytosis. The magnetic complex micelles are further coated with DCA-grafted sheddable PEG-PLL (sPEG) copolymers to generate sPEG/HA/CSO-SS-Hex/Fe_3_O_4_/GA micelles. The charge-reversible property would enable sPEG to shield cationic HA/CSO-SS-Hex/Fe_3_O_4_/GA core at physiological pH, but switch to positive charge in acidic tumor microenvironment and shed off to re-expose HA for CD44 tumor cell targeted and promoting GA delivery.

We characterized the micelle for surface morphology, particle size distribution, drug loading efficiency, stability, pH-sensitivity, redox-sensitivity, magnetic property, drug release and evaluation of pharmacodynamics *in vitro* and *in vivo*. The results showed that the sPEG/HA/CSO-SS-Hex/Fe_3_O_4_/GA micelles successfully accumulate in tumor tissue and be selectively uptake by tumor cells with the help of sPEG charge reversal, magnetism-enhanced EPR and CD44-receptor-mediated endocytosis. The micelle achieved rapid disassembly once internalized into tumor cells. Both *in vitro* and *in vivo* model, this magnetic complex micelle was comprehensively characterized, and its efficacy was evaluated better compared to free GA.

All results indicated that the sPEG/HA/CSO-SS-Hex/Fe_3_O_4_/GA micelle was a suitable complex micelle for targeted drug delivery and intelligent drug release. In addition, we further confirmed that the micelle was an effective targeting system for transporting GA to triple-negative breast cancer with enhanced efficiency.

## Materials and methods

### Chemicals and reagents

Chitosan oligosaccharide (CSO, the average molecular weight =5 kDa, degree of acetylation >80%) (Rekha & Sharma, 2015) was purchased from the Dibai Chemical Reagent Co., Ltd. (Shanghai, China). 1-Hexadecanol (≥99.0%) and N,N′-dicyclohexylcarbodiimide (DCC, ≥95%) was obtained from Sinopharm Chemical Reagent Co., Ltd. (Shanghai, China); 3,3′-dithiodipropionic acid (DTDPA, 99%), 1-ethyl-3-(3-dimethylaminopropyl) carbodiimide hydrochloride (EDCI, 98%), N-hydroxysuccinimide (NHS, 98%), 4-dimethylaminopyridine (DMAP, 99%) and fluorescein isothiocyanate (FITC, 95%), 2,4,6-trinitro-benzenesulfonicacid (TNBS), 1,10-phenanthroline (99%), glutathione (Reduced, 95%), Triethylamine and triphosgene were purchased from the Aladdin Industrial Corporation (Shanghai, China). Methoxypolyethylene glycols（MEO-PEG-OH, the average molecular weight =2 kDa）was obtained from TCI development Co., Ltd (Shanghai, China). Methanesulfonyl chloride was purchased from Shandong Western Asia Chemical Industry Co., Ltd. N6-Cbz-L-Lysine was purchased from Shanghai Shuya medical science and Technology Co., Ltd. Hydrogen bromide (HBr, 33 wt% in acetic acid) was obtained from Bailingwei reagent co., ltd.

Hyaluronic acid (HA, average molecular weight =14600) was obtained from Zhenjiang Dong Yuan Biotech Co., Ltd. (Zhenjiang, China). Gambogic acid (GA) (GA, >97%) was prepared by the laboratory of China Pharmaceutical University (Department of Natural Medicinal Chemistry). Iron (III) acetylacetonate (Fe (acac)_3_) (≥97%), oleylamine (80 ∼ 90%), and 1,2-dodecanediol (≥90%) were obtained from Energy Chemical (Shanghai, China).

1,10-Dioctadecyl-3,3,3,3-tetramethyl indotricarbocyanine iodide (DiR), near-IR lipophilic carbocyanine dye, whose absorption and emission wavelengths were 748 nm and 780 nm obtained from Biotium Inc. (Hayward, CA). Dulbecco’s modified Eagle’s medium (DMEM), 10% fetal bovine serum (FBS) and trypsin containing EDTA were purchased from the Keygen Biotech Corp., Ltd, (Nanjing, China). All organic solvents used were of chromatographic grade or analytical grade.

### Cell lines and animals

Mouse mammary breast tumor 4T1 (Guo et al., 2014) cell line were obtained from were obtained from the Shanghai Institutes for Biological Sciences (China). Cells were cultured in DMEM medium supplemented with 10% FBS in a 37 °C, 5% CO_2_ incubator. Female BALB/c mice (18 ± 2 g) and Sprague Dawley male rats (180–200 g) bought by Qinglongshan animal breeding farm (Nanjing, China). All animals are Specific Pathogen Free (SPF) and feeding at least one week before the experiment.

### Polypeptide copolymers (sPEG) synthesis and characterization

**Scheme 2.**
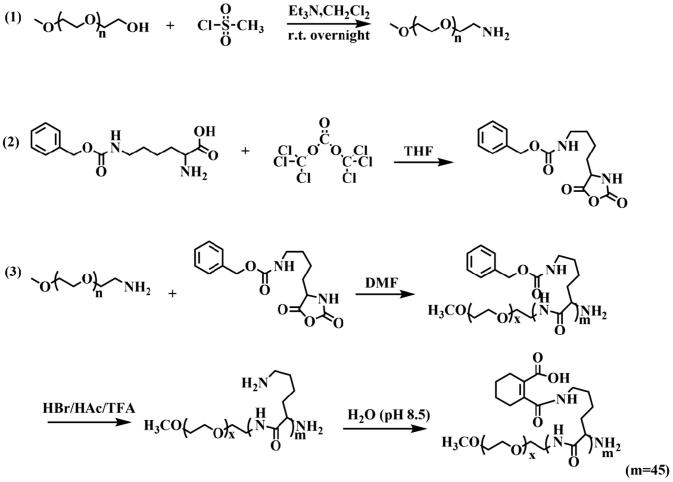
Synthesis route of sPEG copolymer.

The synthesis of sPEG (PEG-PLL (DCA) was according to the Scheme 2 (Liu et al., 2017). Firstly, the synthesis of methoxypolyethylene glycol amine (mPEG-NH_2_, MWCO =2 kDa): MEO-PEG-OH (average MWCO =2 kDa, 2 g, 1 mmol) was dissolved in 20 mL anhydrous toluene and azeotropically distilled under a nitrogen atmosphere at 140 °C for 3 h. After cooling to room temperature, the flask was placed in an ice bath and anhydrous dichloromethane (approximately 4 mL) was gradually added until the solution became clear. Triethylamine (0.208 mL, 1.5 mmol) was added dropwise with stirring, followed by the dropwise addition of mesyl chloride (0.116 mL, 1.5 mmol). After 18 h, the reaction solution was filtered to remove the insoluble white triethylamine hydrochloride salt, followed by precipitation into excess diethyl ether. The off-white product was isolated by filtration and dried under vacuum. Then, 25% aqueous ammonia solution (20 mL) adding to the off-white product, and the reaction solution was stirred for four days at 20 °C. The ammonia was allowed to evaporate slowly over three days at the back of a fume hood. NaOH (5 M) was added dropwise to the solution until the pH reached 13 and the polymer was extracted into dichloromethane (3 × 10 mL). The combined organics were washed with brine and subsequently dried over anhydrous magnesium sulfate. After concentrating under vacuum, the crude PEG-NH_2_ product was precipitated into excess diethyl ether and dried under vacuum to produce PEG-NH_2_.

Secondly, the synthesis of ε-benzyloxycarbonyl-L-lysine (Lys(Z)-NCA) (Daly & Poché, 1988): N6-Cbz-L-Lysine (2 g) and triphosgene (2.0552 g) (1.3 eq:1) were added to anhydrous THF and stirred in oil under a nitrogen atmosphere at 50 °C. After 5 h reaction, the reaction mixture was poured into anhydrous cyclohexane (120 mL), and the resulting suspension stored at −20 °C overnight to assure complete crystallization. The white crystal was dissolved in 50 mL anhydrous ethyl acetate and recrystallization three times according to the above method. Finally, we obtained needle crystal after concentrating under vacuum.

Finally, the synthesis of PEG-PLL (DCA): mPEG-NH_2_ (0.2 g) and Lys(Z)-NCA (1.53 g) were dissolved in 50 mL dried DMF, and stirred under N_2_ at 30 °C for 72 h, after purification, then obtained PEG-PLL(Z). The Z groups were then removed from PEG-PLL(Z) using hydrogen bromide (33 wt.% solution in acetic acid, HBr: PLL =5 eq:1) reaction for 20 min (Zheng et al., 2013; Yi et al., 2016). DCA was then gradually added into PEG-PLL (10 mg/mL, DCA: PEG-PLL =13 eq:1) solution and stirred at 25 °C for 2 h, followed by incubation at pH 8.5 by adding 3 M NaOH for another 2 h. The crude product was then dialyzed against ddH_2_O and freeze dried to obtain DCA-grafted sPEG.

^1^H NMR spectra of the sPEG were recorded by Avance-III 400 spectrometer (Bruker, USA) operated at 500 MHz using D_2_O as solvent.

### sPEG/HA/CSO-SS-hex/Fe3O4/GA preparation and characterization

The method of the preparation of Fe_3_O_4_-loaded CSO-SS-Hex micelles was as previously reported (Sang et al., 2018). GA was transferred into the magnetic micelles using a dialysis technique. Briefly, 15 mg GA was dissolved in 1.5 mL anhydrous ethanol, and the GA ethanol solution was added to the solution of magnetic micelles drop-by-drop under vigorous stirring for 15 min at room temperature. The mixture was ultra-sonicated for 18 min in an ice-bath using a probe-type ultrasonicator (VCX-500; SONICS&MATERIALS, INC, USA) at 150 W followed by dialysis against DDW for 12 h using a dialysis bag (MWCO =1 kDa) and obtained the crude product solution of CSO-SS-Hex/Fe_3_O_4_/GA. The sPEG and HA decorated GA-loaded micelle (sPEG/HA/CSO-SS-Hex/Fe_3_O_4_/GA) was prepared by slowly dropping 3 mL hyaluronic acid solution (1 mg/mL) into the micellar solution of CSO-SS-Hex/Fe_3_O_4_/GA under vigorous stirring followed by sPEG (5 mL, 2 mg/mL), the same method as HA. Then dialysis against DDW for 12 h using a dialysis bag (MWCO =1 kDa). Afterwards, the sPEG/HA/CSO-SS-Hex/Fe_3_O_4_/GA solution were removed from the dialysis bag and filtered through a 0.45 μm membrane to remove large aggregates and unloaded GA.

The drug loading (DL) and entrapment efficiency (EE) (Sang et al., 2018) of GA in the micelle was detected using an HPLC system (Hitachi Primaide System, Japan) with UV detection at 360 nm using a Hitachi Lachrom C_18_ column (5 μm particle size, 4.6 mm I.D × 150 mm) and the mobile phase was a 90/10 (v/v) mixture of methanol and 0.1% formic acid water with a flow rate of 1.0 mL/min for 20 min. The content of Fe_3_O_4_ loaded in micelles was determined according to the previous report (Hauser et al., 2016; Sang et al., 2018).

The nanoparticles were incubated in PBS buffer (0.1 M) at pH 7.4 or 6.5 ± 10 mM GSH at 37 °C to assess the redox and pH-responsiveness of sPEG/HA/CSO-SS-Hex/Fe_3_O_4_/GA micelles. The particle sizes were observed at different time points using Transmission electron microscopy (TEM, Tecnai-12, Philips Company, Holland) and all samples were negatively stained with 0.1% phosphotungstic acid. The zeta potential of different nanoparticles was monitored using dynamic light scattering (Nano-ZS90, Malvern Instruments, U.K) at 25 °C and a scattering angle 90°.

### GA-loaded magnetic complex micelles stability

The dispersion stability of the GA-loaded magnetic complex micelle was investigated by suspending samples at a concentration of 1 mg/mL in phosphate buffer saline (0.01 M PBS, pH =7.4) and FBS. The changes in micelle size were continuously monitored by DLS (Malvern Zetasizer Nano-ZS90, Malvern Instruments, U.K.) for 12 h.

### *In vitro* drug release assay

The *in vitro* drug release profiles were studied by dialyzing 2 mL of the GA-loaded magnetic complex micelle suspension (concentration of micelle was 1.0 mg/mL) in a dialysis bag (MWCO =3500 Da) with 100 mL of PBS buffer (0.01 M) at pH 7.4 or 6.5 ± 10 mM GSH at 37 °C under gentle shaking (100 rpm). At selected time intervals: 0, 1, 2, 4, 8, 12, 24 and 48 h, 50 μL of in bag medium was withdrawn and replaced with an equal volume of fresh in bag medium (Kang et al., 2017). The collected samples were extracted by 250 μL methanol and analyzed by HPLC as described above to determine the amount of released GA. GA release from stock solution was used as a control.

### Hysteresis loop measurement

The magnetization data for Fe_3_O_4_ and sPEG/HA/CSO-SS-Hex/Fe_3_O_4_/GA were determined using a vibrating sample magnetometer (VSM, VSM-175, China) at room temperature (300 K). The applied magnetic field was varied from 1.5 T to −1.5 T to generate hysteresis loops. The magnetic responsiveness of the sPEG/HA/CSO-SS-Hex/Fe_3_O_4_/GA micelles in aqueous solution was tested by simply placing a magnet near the glass vial. A cylindrical sintered N38 NiCuNi-Fe magnet (d = 15 mm, h = 6 mm; the field strength is approximately 0.2 T) was used.

### Cell uptake and intracellular release

For cellular uptake and intracellular release of cargo from the magnetic complex micelles studies, CSO-SS-Hex was labeled with FITC *via* the amino group of CSO and the isothiocyanate group of FITC (Bauhuber et al., 2009; Sang et al., 2018) and the fluorescence probe Nile red (NR) was loaded into micelles in accordance with the protocol for the preparation of the GA-loaded micelles (0.3%, W/W) (Sang et al., 2018). The FITC-labeled sPEG/HA/CSO-SS-Hex/Fe_3_O_4_/NR (FITC-sPEG/HA/CSO-SS-Hex/Fe_3_O_4_/NR), HA/CSO-SS-Hex/Fe_3_O_4_/NR (FITC-HA/CSO-SS-Hex/Fe_3_O_4_/NR) and CSO-SS-Hex/Fe_3_O_4_/NR (FITC-CSO-SS-Hex/Fe_3_O_4_/NR) were prepared following the method for preparing sPEG/HA/CSO-SS-Hex/Fe_3_O_4_/GA and CSO-SS-Hex/Fe_3_O_4_/GA micelles; however, all CSO-SS-Hex was replaced by FITC-CSO-SS-Hex and GA was replaced by NR.

The 4T1 cells were transferred and seeded into a 6-well plate at 1.0 × 10^5^ cells per well and allowed to grown overnight at 37 °C in a humidified 5% CO_2_ atmosphere. The culture medium was replaced with 2 mL fresh medium containing different micelles (FITC-sPEG/HA/CSO-SS-Hex/Fe_3_O_4_/NR, FITC-HA/CSO-SS-Hex/Fe_3_O_4_/NR and FITC-CSO-SS-Hex/Fe_3_O_4_/NR). After incubating at 37 °C for 2 h, the culture medium were removed and washed twice with cold PBS to remove micelles not ingested by the cells; next, the cellular uptake and intracellular release of magnetic complex micelles from cells was visualized using an laser scanning confocal microscopy (Carl Zeiss LSM700, Carl Zeiss AG, Germany) 4T1 cells were incubated with pH 7.4 or 6.5 DMEM to assess the pH-responsiveness of different micelles, and in order to investigate the specific uptake of micelles via HA-receptor mediated endocytosis, the 4T1 cells with 10 mg/mL HA for 1 h before the FITC-sPEG/HA/CSO-SS-Hex/Fe_3_O_4_/NR micelle was added (HA incubated before and no incubate are HA + and HA-, respectively). The FITC and NR labeled CSO-SS-Hex/Fe_3_O_4_/NR micelles were also used as a control to compare with HA/CSO-SS-Hex/Fe_3_O_4_/NR micelles to investigate the influence of the addition of HA on cell uptake.

In order to investigate the 4T1 cells uptake of different micelles, the flow Cytometric Analysis were studied by FACS analysis (Sata et al., 2007; Kim et al., 2008). Also using FITC labeled sPEG/HA/CSO-SS-Hex/Fe_3_O_4_/NR (FITC-sPEG/HA/CSO-SS-Hex/Fe_3_O_4_/NR), HA/CSO-SS-Hex/Fe_3_O_4_/NR (FITC-HA/CSO-SS-Hex/Fe_3_O_4_/NR) and CSO-SS-Hex/Fe_3_O_4_/NR (FITC-CSO-SS-Hex/Fe_3_O_4_/NR) micelles. All flow cytometry was done using a MACS Quant flow cytometer (Miltenyi Biotec, Germany). Data analysis was done using FlowJo software v10 (Ashland, OR).

### *In vitro* magnetic target assay

4T1 cells were at a density of 5 × 10^5^ cells per well in a 100 mm petri dish and maintained in 7 mL 10% FBS of DMEM medium supplemented. After incubation for 24 h in a humidified incubator (37 °C, 5% CO_2_), 100 μg/mL of sPEG/HA/CSO-SS-Hex/Fe_3_O_4_/NR micelle was added. To evaluate the magnetic targeting properties of the magnet micelle, a magnet (Dimension: d = 10 mm, h = 3 mm; field strength approximately 0.2 T) was placed against the outer bottom wall of the petri dish, and the cells were incubated for an additional 3 h. Cells were washed three times with phosphate-buffered saline (PBS), and Nile red fluorescence was analyzed using an OLYMPUS IX53 reflected fluorescence microscope. For the Prussian blue staining experiment, the cells were treated with 4% glutaraldehyde, and then incubated for 30 min at 37 °C with 2 mL prussian blue solution containing 2% HCl solution and 2% potassium ferrocyanide solution (II) trihydrate. After the cell were washed three times with PBS, the Prussian blue staining was analyzed by a OLYMPUS IX53 reflected fluorescence microscope.

### Pharmacokinetic evaluation

Pharmacokinetic studies were performed on Sprague Dawley female rats (180–200 g) free diet for a week. The rats were divided into two groups (sPEG/HA/CSO-SS-Hex/Fe_3_O_4_/GA and GA) and the drugs were administered to mice by caudal vein. The blood samples were collected at different time intervals (0.033, 0.083, 0.167, 0.333, 0.5, 1, 2, 4, 8, 12 and 24 h) in Eppendorf tubes containing di-sodium salt of ethylenediaminetetraacetic acid as an anticoagulant and plasma samples were separated by centrifugating the samples at 8000 rpm (SCILOGEY D3024R centrifuge, SCILOGEY, American) for 10 min. Then, the plasma was added 0.5 M HCl immediately and the samples were stored at −80 °C until extracted with acetonitrile were prepared to analyzed with high-performance liquid chromatography (HPLC) coupled with tandem mass spectrometry (MS/MS) by Shimadzu LCMS-8050 (Shimadzu, Japan). Different pharmacokinetic parameters such as *C*_max_ (the maximum plasma concentration), *t*_1/2_ (elimination half-life), CL (clearance), AUC (area under the concentration time curve) and MRT (mean residence time) were calculated by the software of DAS (drug and statistics, Version 2.0).

### Tumor model

BALB/c female mice (18–20 g, 5 weeks old), were kept under standard laboratory conditions in 12-h light/dark cycle at 25 °C, and given ad libitum access to food and water. The abdominal area of each rat was shaved before the experiment and ensure all the rats no any skin damage that may occur during shaving. Briefly, 4T1 cells (1 × 10^6^) were suspended in 100 mL PBS and subcutaneously injected to the right flank breast of the mice (Luo et al., 2015; Liu et al., 2017). Tumors were allowed to grow to an average volume of 400 mm^3^ in diameter before the experiment.

### *In vivo* imaging

The fluorescent dye DiR was entrapped into the sPEG/HA/CSO-SS-Hex/Fe_3_O_4_ micelles to investigate the tumor-targeting efficacy *in vivo*. The mice were randomly divided into two groups (6 mice per group): One group was not exposed to magnetic attraction for 24 h, whereas the other group was exposed to magnetic field (MF) respectively. Then, the sPEG/HA/CSO-SS-Hex/Fe_3_O_4_/DiR micelles solution were injected into tumor-bearing mice via the tail vein at a DiR concentration of 0.1 mg/kg and stick a magnet (approximately 0.2 T) on the top of the tumor by tape. At 0.083, 0.5, 1, 2, 4, 8 and 24-h postinjection, the mice were anesthetized, and whole-body fluorescence images were acquired using a nearinfrared fluorescence imaging system (Kodak, Rochester, NY) with a wavelength set at *E*x =748 nm and *E*m =780 nm. During the imaging acquiring process, 3% isoflurane anesthesia (Abbott laboratories, Chicago, IL) (Huang et al., 2018) was administered to the mice via a nose cone system as mean ± SD (*n* = 6).

### Anti-tumor activity

15 days after the cell injection, the BALB/c female mice whose xenografts reached a volume of around 400 mm^3^, the mice were randomly divided into three groups (6 mice per group): saline group, GA solution group and sPEG/HA/CSO-SS-Hex/Fe_3_O_4_/GA group; each mouse was injected intravenously via the tail vein with the above formulations at a dose of 6 mg/kg of GA every 2 days for fourth times. The tumor sizes were measured every two days by using a caliper, and the therapeutic results of each group were evaluated by calculating the tumor volumes (formula: tumor volume = length × width × width/2) (Wang et al., 2017a) and the total weight of tumors. Two days after last treatment, tumors were harvested (Liu et al., 2016).

### *In vivo* biosafety evaluation

BALB/c female mice (18–20 g), and were randomly divided into three groups to receive IV injection of ddH_2_O (control group), GA solution (6 mg/kg) and sPEG/HA/CSO-SS-Hex/Fe_3_O_4_/GA (6 mg/kg) every three days, and two days after the third time, organ and blood were collected. To check the *in vivo* organ toxicity of the particles, we performed H&E staining of the major organs of mice. The liver function was evaluated by measuring the serum level of aspartate aminotransferase (AST) and alanine aminotransferase (ALT) using AST and ALT activity Assay Kit (Jian Cheng Biotech, China). The renal function was evaluated by measuring the serum levels of urea nitrogen (BUN) and creatinine (CRE) using colorimetry according to the manufacturer's instruction (Jian Cheng Biotech, China). Major organs (heart, liver, spleen, lung and kidney) were fixed in 4% paraformaldehyde overnight and cut into 5 mm sections for hematoxylin and eosin (H&E) staining, and the histology of different organs was assessed using Olympus microscope.

### Statistical analysis

All statistical analysis was performed using graphpad prism software (Version 5.01). The quantitative data were presented as mean ± standard deviation (SD) or mean ± standard error of mean (SEM).

## Results and discussions

### Synthesis and characterization of polypeptide copolymers (sPEG)

Synthesis route of sPEG was divided into three steps (Scheme 2). The structures of copolymers were characterized by ^1^H NMR. The peaks around 3.36 ppm (a) and 3.68 ppm (b) were attributable to the protons (–OCH_3_) and (–CH_2_–CH_2_) in PEG, and the peaks around 4.21 ppm (e), 2.88 ppm (i) and 1.30-1.68 ppm (f-h) were attributable to the chain protons (–CH) and (–CH_2_) in PLL. The peaks at 1.57 ppm (d) and 2.16 ppm (c) were characteristic signals of DCA (Figure S1), and the grafted efficiency was about 100%. By calculating the ratios of peak areas of a to c or a to d, the polymerization degrees of PLL in sPEG was about 17.5.

**Figure 1.**
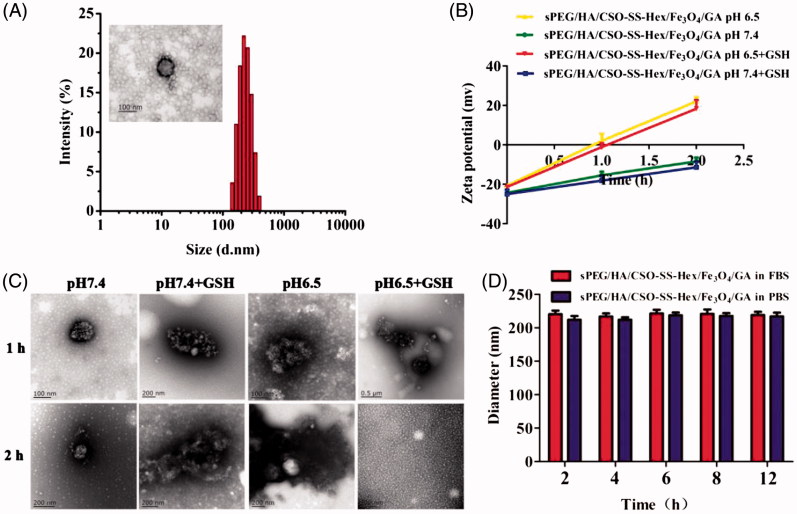
Characterization of sPEG/HA/CSO-SS-Hex/Fe_3_O_4_/GA micelles. (A) Size distribution and TEM of the micelles in water. (B) and (C) zeta potential and TEM of the micelles at pH 7.4 or pH 6.5 ± 10 mM GSH at 37 °C for 1 h and 2 h. (D) The stability of the micelles in PBS and FBS at 37 °C.

### Preparation and characterization of GA-loaded magnetic complex micelles

The hydrophobic GA was easily encapsulated into sPEG/HA/CSO-SS-Hex/Fe_3_O_4_ micelles using a dialysis method. The GA-loaded magnetic complex micelles (sPEG/HA/CSO-SS-Hex/Fe_3_O_4_/GA) has positive charge redox responsive CSO-SS-Hex/Fe_3_O_4_/GA core with surface physical adsorption of negative charge HA and charge-reversible sPEG shell. The GA-loaded magnetic complex micelles had hydrodynamic size of 220 ± 2.3 nm with the zeta potential at about -25.8 mv; however, the zeta potential of HA/CSO-SS-Hex/Fe_3_O_4_/GA was about 15.6 mv. The decreased zeta potential of sPEG/HA/CSO-SS-Hex/Fe_3_O_4_/GA micelles were due to the effective shielding effect of sPEG, which diminished the electrostatic repulsion caused by PLL fragments (Luo et al., 2015). The TEM imaging showed a homogeneous distribution of spherical nanoparticles with a mean diameter at 50–100 nm (Figure 1(A)). The including drug loading (DL) and entrapment efficiency (EE) were 27.5% and 99.8%, and the content of Fe_3_O_4_ loaded in micelles was 17.02% approximately. The loading of Fe_3_O_4_ could improve the DL and EE of GA, which can be attributed to the stronger hydrophobic interactions of GA, the hydrophobic fragment of the copolymer and the Fe_3_O_4_ nanoparticles (Sang et al., 2018).

To evaluate the redox and pH responsiveness of GA-loaded magnetic complex micelle, it was incubated in PBS ± GSH at pH 7.4 or 6.5 for 2 h (Figure 1(B,C)). Despite the presence of GSH, the zeta potential of sPEG/HA/CSO-SS-Hex/Fe_3_O_4_/GA slowly increased from −25.8 mV to −11.2 mV at pH 7.4 (Figure 1(D)), which was attributable to the slow hydrolysis of amide between DCA and PLL at pH 7.4 (Hauser et al., 2016). However, at pH 6.5, the zeta potential of sPEG/HA/CSO-SS-Hex/Fe_3_O_4_/GA significantly increased to 24.6 mV within 2 h (Figure 1(B)), suggesting the shedding of sPEG caused by amide bond degradation at low pH. And the redox/pH dual-responsive of sPEG/HA/CSO-SS-Hex/Fe_3_O_4_/GA micelles were further evaluated by the observation of TEM (Figure 1(C)). The result indicated acid and reducible condition triggered micelles disassembly, which was due to sPEG shedding and the cleavage of disulfide bond crosslinks in the core, thereby destroying the amphiphilic core shell structure of sPEG/HA/CSO-SS-Hex/Fe_3_O_4_/GA micelles.

These results confirmed the charge-reversible property of sPEG, which would be able to shield cationic sPEG/HA/CSO-SS-Hex/Fe_3_O_4_/GA core to at physiological pH to avoid ‘off-target’ GA delivery, but quickly shed off at low pH to re-expose HA/CSO-SS-Hex/Fe_3_O_4_/GA core for tumor-targeted GA delivery. In conclusion, the pH-redox responsiveness of sPEG/HA/CSO-SS-Hex/Fe_3_O_4_/GA micelles would allow maintaining superior colloidal stability in extracellular conditions, whereas facilitating the release of their payload GA in the reducible cytoplasm.

The stability of the sPEG/HA/CSO-SS-Hex/Fe_3_O_4_/GA micelles were also evaluated by dispersion in two commonly used biological media, pH 7.4 PBS and FBS at 37 °C. The result showed that the micelles remained stable with a slight size change but no aggregation over 12 h in both PBS and FBS (Figure 1(D)).

### *In vitro* drug release

**Figure 2.**
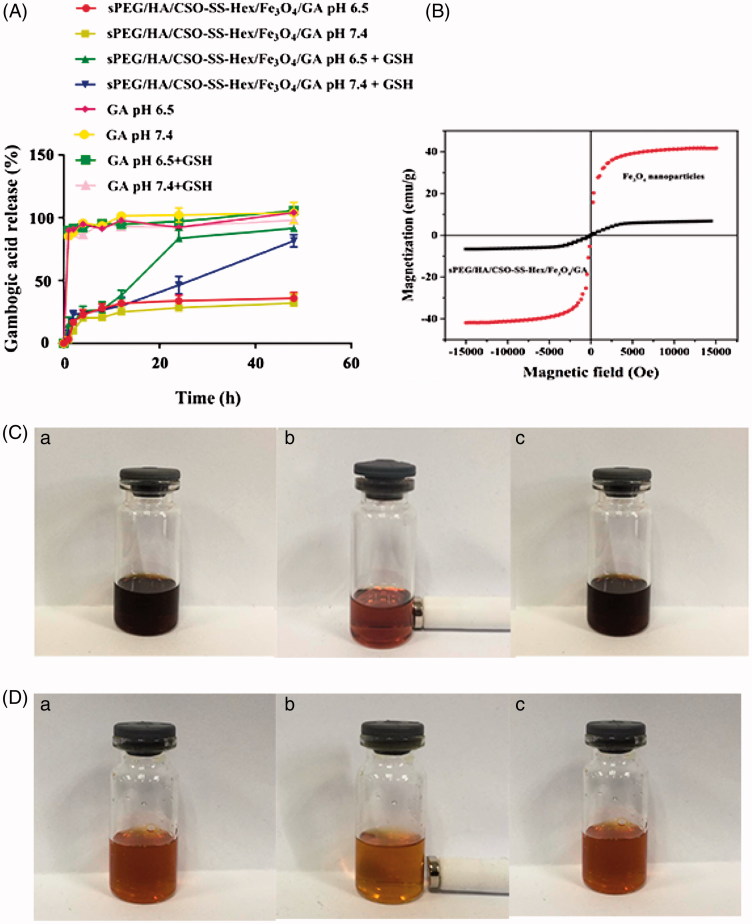
*In vitro* GA release and magnetic properties of sPEG/HA/CSO-SS-Hex/Fe_3_O_4_/GA micelles. (A) *In vitro* release profiles of GA from sPEG/HA/CSO-SS-Hex/Fe_3_O_4_/GA micelles under different simulated conditions at 37 °C (The error bars in the graph represent standard deviation (*n* = 3)). (B) Hysteresis loops of Fe_3_O_4_ nanoparticles and sPEG/HA/CSO-SS-Hex/Fe_3_O_4_/GA micelles solution. (C and D) refer to the Fe_3_O_4_ nanoparticles chloroform solution and sPEG/HA/CSO-SS-Hex/Fe_3_O_4_/GA micelles water solution of (a) before, (b) after imposing an external magnetic field and (c) removal of magnetic fields.

To achieve successful GA-loaded magnetic complex micelles be effectively delivered into tumor, we investigate the influence of GSH and pH on drug-release behavior, we performed GA release experiments with sPEG/HA/CSO-SS-Hex/Fe_3_O_4_/GA in the presence or absence of 10 mM GSH ± PBS at pH 7.4 or 6.5 and 37 °C. As shown in Figure 2(A), without GSH, only a small amount of drug about 30% was released from the micelles at both pH 6.5 and pH 7.4. The presence of GSH significantly increased

GA release up to about 85% at different pH within 48 h, which was due to CSO-SS-Hex/Fe_3_O_4_/GA micelle disassembly. As a control, free GA was completely released within 10 h in different condition. Therefore, the redox/pH dual-responsive of sPEG/HA/CSO-SS-Hex/Fe_3_O_4_/GA would prevent GA leakage in the extracellular environment, but facilitate GA release at the intracellular bioreducible condition, which enhance the micelles tumor therapeutic efficacy.

### Magnetic properties of sPEG/HA/CSO-SS-hex/Fe_3_O_4_/GA

The magnetization curves of Fe_3_O_4_ nanoparticles and sPEG/HA/CSO-SS-Hex/Fe_3_O_4_/GA micelles displayed in Figure 2(B) were investigated on a VSM system at room temperature. These particles did not exhibit obvious hysteresis in a low magnetic field, indicating that these particles show superparamagnetism (Lee et al., 1996). The saturation magnetization of the magnetic sPEG/HA/CSO-SS-Hex/Fe_3_O_4_/GA complex micelle was 7.99 emu/g, considerably less than that of Fe_3_O_4_ nanoparticles (41.23 emu/g). The loss of magnetization should be attributed to the presence of the copolymer coating on the surface of Fe_3_O_4_ nanoparticles. The magnetic responsive of the Fe_3_O_4_ nanoparticles and sPEG/HA/CSO-SS-Hex/Fe_3_O_4_/GA complex micelles in solution were visualized by a simple experiment in which a 0.2 T magnet was placed near the glass vials (Figure 2(C,D)). The sPEG/HA/CSO-SS-Hex/Fe_3_O_4_/GA complex micelles could concentrate toward the wall adjacent to the magnet in a short time, and the solution color faded. When removal of the magnetic fields, the complex micelles were rapidly re-dispersed into a uniform solution, indicating no agglomeration of micelles, which is the same properties of Fe_3_O_4_ nanoparticles. The magnetic micelles developed here were highly sensitive to an external magnetic field and it was feasible to guide the complex micelles using an external magnetic field; thus, the micelles have potential as a magnetically guided system for drug delivery.

### Cellular uptake and intracellular drug release

**Figure 3.**
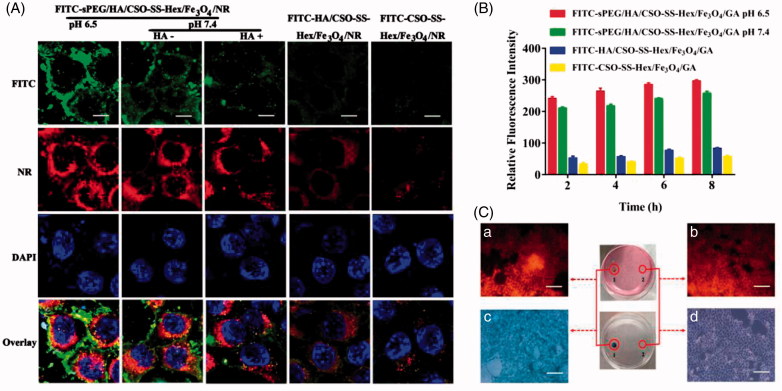
Cell uptake and intracellular drug release of complex micelles in 4T1 cells. (A) 4T1 cells were incubated with FITC and NR-loaded sPEG/HA/CSO-SS-Hex/Fe_3_O_4,_ HA/CSO-SS-Hex/Fe_3_O_4_ and CSO-SS-Hex/Fe_3_O_4_ at different condition for 2 h, and labeled with DAPI (blue) to identify cell nuclei. The bar shown is 20 μm. (B) Quantitative determination of FITC by flow cytometry after 4T1 cells were incubated with FITC-sPEG/HA/CSO-SS-Hex/Fe_3_O_4_/GA micelles at pH 6.5 or 7.4, FITC-HA/CSO-SS-Hex/Fe_3_O_4_/GA and FITC-CSO-SS-Hex/Fe_3_O_4_/GA for 2, 4, 6, 8 h. (C) Micrographs of sPEG/HA/CSO-SS-Hex/Fe_3_O_4_/GA micelles after 3 h incubation in an external magnetic field, (a and b) refer to nile red fluorescent intensity of cells located in circle 1 and circle 2; (c and d) refer to Prussian blue staining of cells located in circle 1 and circle 2. Circle 1 indicates magnet position (targeted area), whereas circle 2 indicates an area with weaker magnetic field strength (control area). The bar shown is 50 μm.

To investigate the cellular uptake and intracellular drug release mechanism of sPEG/HA/CSO-SS-Hex/Fe_3_O_4_/GA, 4T1 cells (overexpressing CD44 receptor) incubated with FITC and NR (Nile red) (Hu et al., 2015) labeled sPEG/HA/CSO-SS-Hex/Fe_3_O_4_/GA, HA/CSO-SS-Hex/Fe_3_O_4_/GA and CSO-SS-Hex/Fe_3_O_4_/GA micelles (FITC-sPEG/HA/CSO-SS-Hex/Fe_3_O_4_/NR, FITC- HA/CSO-SS-Hex/Fe_3_O_4_/NR and FITC-CSO-SS-Hex/Fe_3_O_4_/NR) were observed by fluorescence microscope (Song et al., 2014; Dey et al., 2016). The results showed (Figure 3(A)) that the fluorescent intensity (FITC and NR) of 4T1 cells treated with FITC-sPEG/HA/CSO-SS-Hex/Fe_3_O_4_/NR (HA-) micelles was significantly stronger than those treated with FITC-sPEG/HA/CSO-SS-Hex/Fe_3_O_4_/NR (HA+) micelles, also the FITC- HA/CSO-SS-Hex/Fe_3_O_4_/NR micelles was stronger than those treated with FITC-CSO-SS-Hex/Fe_3_O_4_/NR micelles under the same imaging parameters throughout the cell imaging process. This finding revealed that HA could competitively bind to CD44 receptors against the sPEG/HA/CSO-SS-Hex/Fe_3_O_4_/GA micelles and played a critical role in increasing cell uptake via receptor-mediated endocytosis, which resulting in different fluorescence intensity. The 4T1 cells treated with FITC-sPEG/HA/CSO-SS-Hex/Fe_3_O_4_/NR at pH 6.5 was obviously stronger than those treated with pH 7.4. Hence, the surface assembly of sPEG could prevent GA uptake at a physiological pH, but allow GA-loaded magnetic complex micelles uptake at low pH. All results demonstrated that sPEG/HA/CSO-SS-Hex/Fe_3_O_4_/GA micelles not only specifically bind to CD44 and internalize into cancer cells *via* CD44 receptor mediated endocytosis but also released the drug at low pH.We further investigated the cellular uptake behaviors of the FITC-sPEG/HA/CSO-SS-Hex/Fe_3_O_4_/GA micelles against 4T1 cells in different time of 2, 4, 6, 8 h by flow cytometry (Figure 3(B)). All micelles (FITC-sPEG/HA/CSO-SS-Hex/Fe_3_O_4_/GA, FITC-HA/CSO-SS-Hex/Fe_3_O_4_/GA and FITC-CSO-SS-Hex/Fe_3_O_4_/GA) in 4T1 cells both showed a time-dependent increase in fluorescence intensity. The FITC-HA/CSO-SS-Hex/Fe_3_O_4_/GA micelles showed higher fluorescence intensity than FITC-CSO-SS-Hex/Fe_3_O_4_/GA, which dependent on the role of HA. The micelles in pH 6.5 showed higher fluorescence intensity than pH 7.4, which shows that the GA-loaded magnetic complex micelles is responsive to low pH. All result showed the same conclusion as fluorescence microscope.

### *In vitro* magnetic target assay

An *in vitro* magnetic targeting experiment was designed and performed to examine the magnetic targeting property of the sPEG/HA/CSO-SS-Hex/Fe_3_O_4_/GA micelles. A commercially available magnet (approximately 0.2T) was placed against the outer bottom surface of the petri dish, and cells in the petri dish were referred to as the targeting area (circle 1) and the control area (circle 2). After washed with PBS three times, the cells were investigated for nile red fluorescent intensity and Prussian blue staining. As visualized under the microscope, the result showed that the external magnetic field could significantly increase the local concentration of magnetic micelle, the cells located inside circle 1 could take up considerably more than cells located inside circle 2 after 3 h incubation (see Figure 3(C)(a) vs. Figure 3(C)(b) and Figure 3(C)(c) vs. Figure 3(C)(d)), which implies that the magnetic micelles could efficient carry cargo to the targeted area under magnetic guidance, enabling the selective and effective killing of cells in specific area.

### Pharmacokinetic evaluation of GA and sPEG/HA/CSO-SS-hex/Fe_3_O_4_/GA *in vivo*

The analytical method was validated in our laboratory. The peak area ratio of olmesartan to diazepam (internal standard) in rat plasma was linear with respect to the analyte concentration over the range 0.1–100 μg/mL. For samples preparation protein precipitation method was used. 100 μL plasma (contain 50% 0.5 M HCl) was taken in an Eppendorf tube and spiked with 50 mL of mixed standard solution (200 ng/mL diazepam, different concentration of GA) which dissolved in acetonitrile and a volume of 100 mL of chilled acetonitrile was added as a protein precipitating agent, vortexed for 1 min and then centrifuged at 14,000 rpm for 10 min. The supernatant layer was filtered through 0.45 μm syringe filters and 20 mL of the sample solution was injected for HPLC-MS analysis.

The calibration curve showed a good linearity over concentration ranges (0.01–500 μg/mL) with correlation coefficient (r^2^) of 0.9996 and the standard curve equation is A = 0.7157C + 0.0014. The accuracy was evaluated with low, medium and high concentrations was found to be 96.62, 103.16 and 98.70%, respectively. The intra-day RSD of low, medium and high concentrations of GA solution was also detected as 1.1, 0.8 and 0.7%, respectively. The inter-day RSD was 1.4, 1.2 and 1.3%, respectively, which were <1.5%, indicating that the method is precise, accurate and reliable was found satisfactory and proved to be adequate for the determination of GA in plasma.

**Figure 4.**
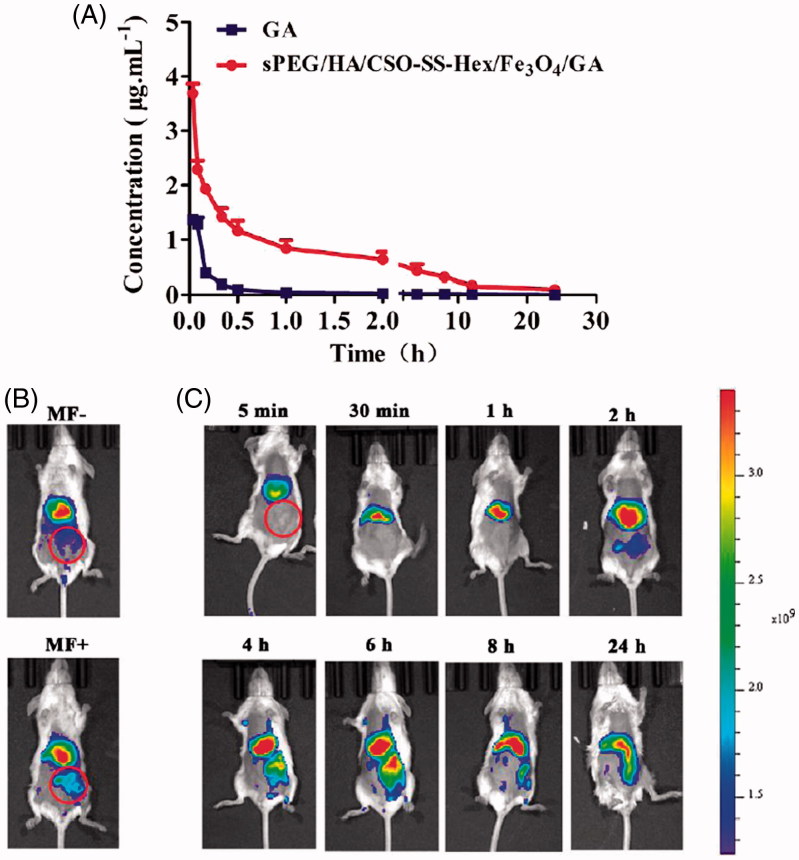
Pharmacodynamics evaluation of sPEG/HA/CSO-SS-Hex/Fe_3_O_4_/GA *in vivo*. (A) Mean blood concentration–time curve of Sprague Dawley female rats after caudal vein administration of sPEG/HA/CSO-SS-Hex/Fe_3_O_4_/GA micelles and free GA (Each value represents the mean ± SD, *n* = 6). (B and C) *In vivo* fluorescence images of tumor-bearing mice acquired after injecting DiR-conjugated sPEG/HA/CSO-SS-Hex/Fe_3_O_4_/DiR micelles suspensions, it showed the tumor not exposing (MF-) or exposing (MF+) to magnetic fields at 24 h (B), while (C) showed the tumor exposing to magnetic fields at different time points (the red circle indicates an area of tumor position).

The plasma concentration profile of GA following the application of sPEG/HA/CSO-SS-Hex/Fe_3_O_4_/GA micelles in wistar rats is shown in Figure 4(A) and the pharmacokinetic parameters showed in Table S1. The *C*_max_ was found to be 3.85 ± 2.34 μg/mL, which was higher than free GA 1.61 ± 0.98 μg/mL because of the instability of GA *in vivo*. The t_1/2_ (0.38 ± 0.20 h) of GA in plasma was noticed after the application of magnetic micelles, which is approximately, almost 4 times higher compared to free GA. This higher value of *C*_max_ and *t*_1/2_ may because of the instability of GA *in vivo*. It was also observed that the AUC _0-t_, AUC _0-∞_ and MRT values of the micelles were all significantly (*p* < .05) higher than the free GA. This could be due to the maintenance of the concentration of the micelles within the pharmacologically effective range for a longer period of time. In summary, the developed GA-loaded magnetic complex micelles system successfully improved the biocompatibility of chemotherapeutic drugs.

### *In vivo* imaging

The tumor-targeting abilities of sPEG/HA/CSO-SS-Hex/Fe_3_O_4_/GA were assessed in the 4T1 breast tumor xenograft mouse model using DiR. DiR was selected as the fluorescent probe in this study could effectively reduce the interference of animal auto-fluorescence according to its NIR excitation and emission wavelength (Sharma & Al, 2006). Time-dependent biodistribution of sPEG/HA/CSO-SS-Hex/Fe_3_O_4_/DiR was visualized after intravenous administration using a noninvasive NIR imaging technique. And the fluorescence detected in sPEG/HA/CSO-SS-Hex/Fe_3_O_4_/DiR was sustained at the tumor location at all the time points of postinjection, which suggest a tumor-specific accumulation of the drug in 24 hours after post-administration. As represented in Figure 4(B), sPEG/HA/CSO-SS-Hex/Fe_3_O_4_/DiR exhibited the fluorescence signal of exposing (MF+) the tumors to magnetic fields was stronger than not exposing (MF-), indicate that the magnetic of the micelles can further enhance the tumor targeting effect.

### Anti-tumor activity of sPEG/HA/CSO-SS-hex/Fe_3_O_4_/GA *in vivo*

After observing the anticancer activities of sPEG/HA/CSO-SS-Hex/Fe_3_O_4_/GA *in vitro* studies, we moved forward to evaluate their antitumor effect in an *in vivo* animal tumor model. The mouse 4T1 cell line was used to establish the TNBC tumor model in BALB/c mice. When subcutaneous 4T1 tumors grew to about 400 mm^3^ in volume, the tumor-bearing mice were divided into three groups (*n* = 6): (i) saline, (ii) free GA, (iii) sPEG/HA/CSO-SS-Hex/Fe_3_O_4_/GA (6 mg/kg of GA). Drugs were intravenously administrated via tail vein into 4T1-bearing mice on day 1, 3, 5, 7 and 9. The therapeutic efficacy was observed by measuring tumor volumes for 11 days.

**Figure 5.**
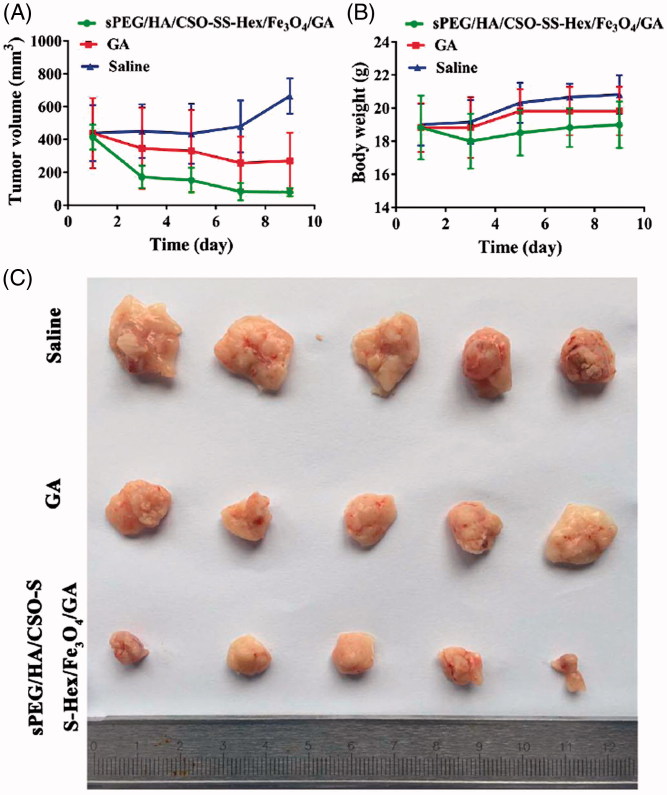
*In vivo* anti-tumor effect of sPEG/HA/CSO-SS-Hex/Fe_3_O_4_/GA. (A) Change of tumor volume in 4T1 cell-bearing mice after intravenous injection of different formulations. The two groups of GA and sPEG/HA/CSO-SS-Hex/Fe_3_O_4_/GA all showed the extremely significant difference (*p* < 0.01) vs. saline control. (B) Change of body weight in 4T1-bearing mice during the study. (C) Images of excised tumors of all groups at the end of study. (11 days post the initiation of treatment). Points are presented as mean ± SEM (*n* = 6).

As controls, the tumor volumes of saline gradually increased to 792 mm^3^ at day 11, showing a minor tumor inhibition effect of free GA (Figure 5(A)). The sPEG/HA/CSO-SS-Hex/Fe_3_O_4_/GA treatment exhibited the strongest effect in inhibiting tumor growth. The tumor suppression rate (TSR) of GA and sPEG/HA/CSO-SS-Hex/Fe_3_O_4_/GA were 38.43% and 84.1%, respectively. Tumors were collected at the end of study (Figure 5(C)). In particular, the tumor volumes in the sPEG/HA/CSO-SS-Hex/Fe_3_O_4_/GA group were extremely smaller than those of GA solution group (*p* < .01), indicating a statistically improved antitumor effect of sPEG/HA/CSO-SS-Hex/Fe_3_O_4_/GA compared to free GA group. Moreover, throughout the therapeutic course, there was no obvious body weight loss in all the groups, suggesting the safety of the GA and sPEG/HA/CSO-SS-Hex/Fe_3_O_4_/GA (Figure 5(B)).

### Biosafety of sPEG/HA/CSO-SS-hex/Fe_3_O_4_/GA *in vivo*

**Figure 6.**
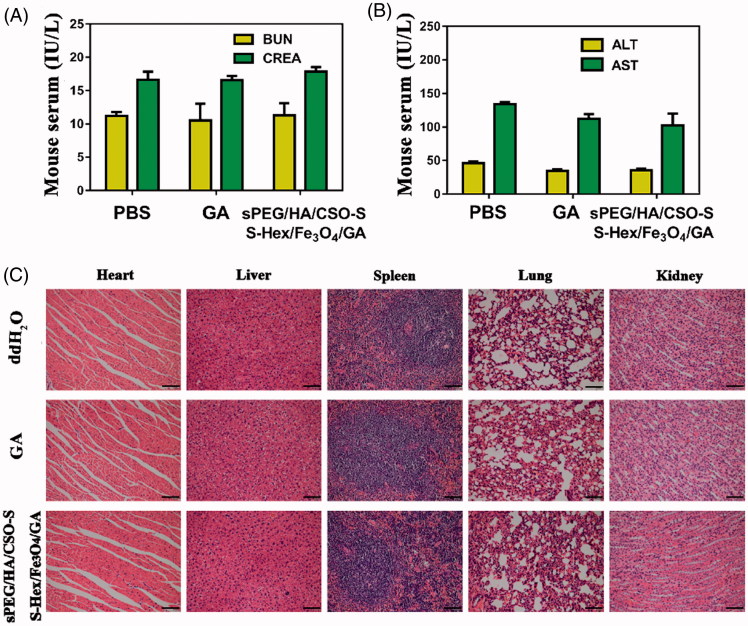
Biosafety of sPEG/HA/CSO-SS-Hex/Fe_3_O_4_/GA micelles *in vivo*. (A) Serum levels of BUN and CRE (renal function) at 48 h after last treatment. (B) Serum levels of ALT and AST (liver function) at 24 h after last treatment. (C) H&E staining of female rat organs (hearts, livers, spleens, lungs, and kidneys) at the end of experiments. The bar shown is 50 μm.

In the present study, serum BUN/CRE and serum ALT/AST were measured at the end of the experiment, which are critical biomarkers of renal and liver damage, respectively. The results showed that serum BUN/CRE and serum ALT/AST were not significantly different among all groups (Figure 6(A,B)), indicating no significant renal or liver toxicity of sPEG/HA/CSO-SS-Hex/Fe_3_O_4_/GA micelles.

The potential toxicity of sPEG/HA/CSO-SS-Hex/Fe_3_O_4_/GA micelles were further investigated in vital organs using H&E staining. After 6 mg/kg GA dosages of treatment, mouse organs including heart, liver, spleen, lung and kidney did not show histopathological abnormalities, lesions or degenerations in all three groups, confirming the caudal vein administration of sPEG/HA/CSO-SS-Hex/Fe_3_O_4_/GA micelles was well tolerant and bio-compatible. Clearly, there are no histological changes observed in the major organs including liver, lung, spleen, kidney, and heart at half a month post-injection, compared with the PBS control (Figure 6(C)). This suggests that the designed GA-loaded magnetic complex micelles show good biocompatibility *in vivo*.

## Conclusions

The present work reported the successful application of sPEG and HA coated redox/pH dual-responsive magnetic complex micelle (sPEG/HA/CSO-SS-Hex/Fe_3_O_4_/GA) as a drug carrying system to simultaneously deliver a natural compound GA to improve curative effect of TNBC. The micelles prevent the cellular uptake of GA at pH 7.4 but improved uptake of the drugs in TNBC cells at lower pH 6.5 both *in vitro* and *in vivo*, resulting in more efficient combinatorial antitumor effect. The magnetic complex micelle was highly stable under physiological conditions but was quickly dis-assembled to release drug loads in the presence of 10 mM GSH with the help of the magnetism-EPR and was selectively taken up into tumor cells *via* HA-receptor-mediated endocytosis. Rapid disassembly was achieved once internalized into tumor cells, thereby improving intracellular drug release and increasing antitumor efficacy. In summary, from the observations in this study, it is suggested that sPEG/HA/CSO-SS-Hex/Fe_3_O_4_/GA with excellent bioavailability, controllable release and multi-functionality is an effective and promising strategy for TNBC chemotherapy.

## Supplementary Material

Supporting_Information.pdf
