# Supplementary material for: A novel redox/pH dual-responsive and hyaluronic acid-decorated multifunctional magnetic complex micelle for targeted gambogic acid delivery for the treatment of triple negative breast cancer
Source: Drug Deliv. 2018 Oct 18;25(1):1846–57. doi: 10.1080/10717544.2018.1486472 (PMC6225507; doi:10.1080/10717544.2018.1486472)
Supplement: Supporting_Information.pdf [file IDRD_A_1486472_SM6912.pdf]

Supporting Information for "A novel redox/pH dual-responsive and hyaluronic acid-decorated multifunctional magnetic complex micelle for targeted gambogic acid delivery for the treatment of triple negative breast cancer"

Mangmang Sang,[1,2] Fulei Liu,[3] Yang Wang,[1,2] Renjie Luo,[1,2] Xiaoxian Huan,[3] Lingfei Han,[1,2] Zhongtao Zhang,[3] Feng Feng,[3] Wei Qu,[3] Wenyuan Liu,[1,2] & Feng Zheng[1,2]

[1] *Key Laboratory of Drug Quality Control and Pharmacovigilance, Ministry of Education, China Pharmaceutical University, Nanjing 210009, People's Republic of China*
[2] *Department of Pharmaceutical Analysis, China Pharmaceutical University, Nanjing 210009, People's Republic of China*
[3] *Department of Natural Medicinal Chemistry, China Pharmaceutical University, Nanjing 210009, People's Republic of China*

[Corresponding author] Wenyuan Liu: Prof., Tel/Fax: +86 025 83271038; E-mail address: liuwenyuan8506@163.com. Feng Zheng: Prof., Tel: +86 13913983206; E-mail: cpu_analyst@126.com

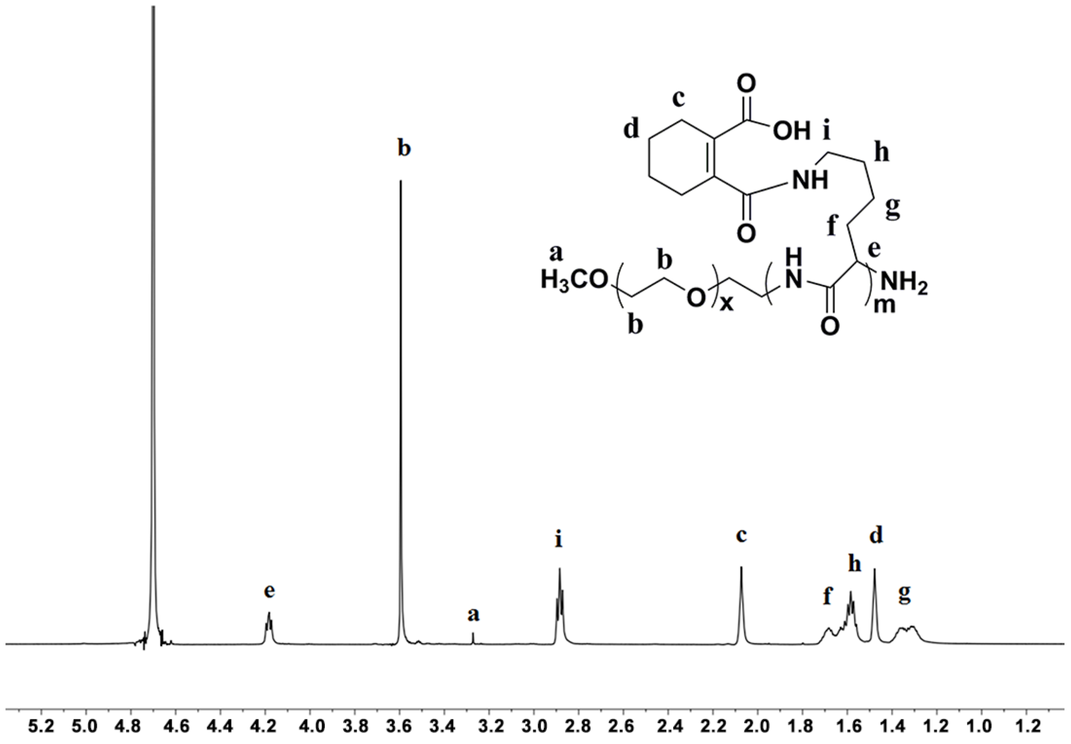

Figure S1. The ^{1}H NMR spectra of sPEG in D_2O.

Table S1. Pharmacokinetic parameters of sPEG/HA/CSO-SS-Hex/Fe_3O_4/GA and GA (n=6).

Parameter	Units	sPEG/HA/CSO-SS -Hex/Fe_3O_4/GA	GA
C_{max}	μg/ml	3.85 ± 2.34	1.61 ± 0.98
$t_{1/2}$	h	0.38 ± 0.20	0.09 ± 0.01
CL	(mg/kg)/(μg/ml)/h	4.14 ± 1.23	23.98 ± 4.56
$AUC_{0\text{-}t}$	μg/ml*h	1.44 ± 1.08	0.25 ± 0.11
$AUC_{0\text{-}\infty}$	μg/ml*h	1.45 ± 0.78	0.25 ± 0.14
MRT	h	0.49 ± 0.22	0.14 ± 0.23
